# “*Mens Sana In Corpore Sano*”: Exercise and Hypothalamic ER Stress

**DOI:** 10.1371/journal.pbio.1000464

**Published:** 2010-08-24

**Authors:** Pablo Blanco Martínez de Morentin, Miguel López

**Affiliations:** 1 Department of Physiology, School of Medicine, University of Santiago de Compostela-Instituto de Investigación Sanitaria, Santiago de Compostela, Spain; 2 CIBER Fisiopatología de la Obesidad y Nutrición (CIBERobn), Santiago de Compostela, Spain

## Abstract

A novel mechanism explains how exercise exerts its beneficial effects on energy balance through an effect at the level of the hypothalamus.

Exercise is a mainstay recommendation for fending off obesity and preventing diabetes, cardiovascular problems, or simply to release stress. Although physicians are certain about the many health benefits of exercise, especially for obese patients, a satisfying explanation for why exercise leads to long-term improvements in health profiles isn't nearly as clear. More obviously, exercise can tip the positive imbalance between energy acquisition and energy expenditure (Figure 1) [1]–[5] that can eventually result in obesity. In Western countries, levels of obesity and its related metabolic disorders are increasing at a rate that is considered of epidemic proportions. Although the increasing prevalence of obesity is anticipated by a combination of genetic predisposition and social and environmental factors, working out the precise contributions is fundamental to understanding the basic molecular mechanisms controlling energy balance.

**Figure 1:**
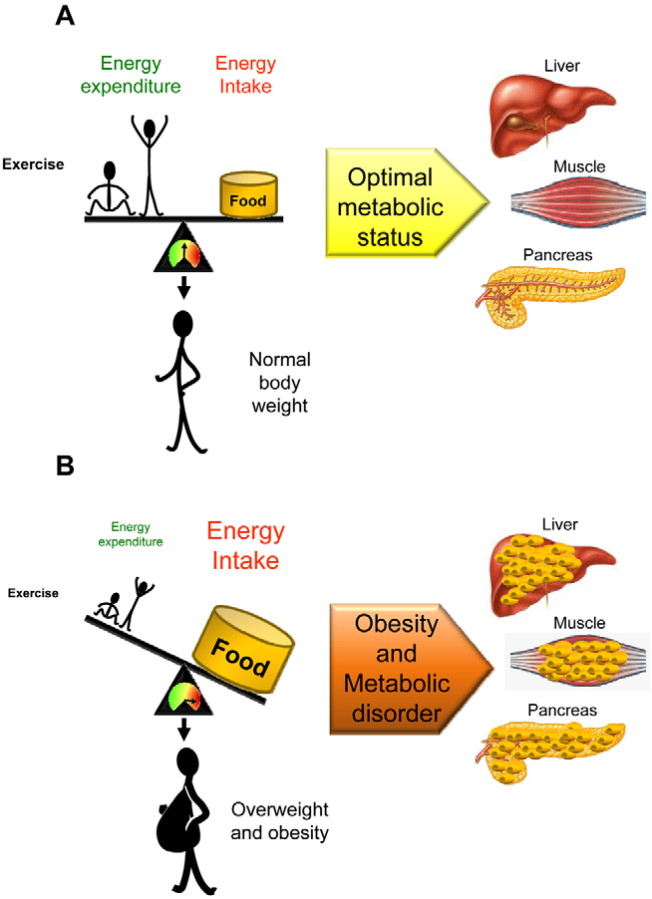
Altered energy balance leads to obesity and metabolic disorders. (A) When the quantity of energy absorbed by an animal (i.e., food intake) equals its energy expenditure (i.e., physical activity/exercise), the result is a neutral energy balance that permits body weight stability. In this situation lipids are stored in white adipose tissue (WAT). (B) An imbalance in either food intake or energy expenditure leads to increased body weight and obesity. In this context, the storage capacity of WAT may become saturated, which redirect lipids to be accumulated in peripheral organs such as liver, muscle, and pancreas. In a first step, these lipids are accumulated as triacylglycerols (TGs). When the storage capacity of these tissues is also saturated, excess of lipids, enters in alternative non-oxidative pathways that results in production of toxic reactive lipid species (such as diacylglycerols and ceramides) leading to tissue-specific damage, a process known as lipotoxicity.

At a biochemical and physiological level, there is a considerable amount of cross-regulation and integration between the mechanisms controlling food intake, energy expenditure, and fat deposition. Interestingly, increased fat deposition and excessive body weight are associated with allostatic changes aiming to restore energy balance. For instance, increased fat mass is associated with increased production of leptin and other adipose-derived hormones directed to reduce food intake and promote energy expenditure. Conversely, states of reduced food intake, such as fasting, induce an allostatic response directed to save energy stores and increase the drive for food intake. However, despite these allostatic responses to maintain energy balance, under conditions of chronic hypercaloric excess (e.g., overnutrition) and/or impedance (e.g., reduced physical exercise) the efficiency and accuracy of these regulatory mechanisms are defective. It has been suggested that toxic effects of lipid accumulation in peripheral tissues, such as pancreatic β cells, liver, heart, and skeletal muscle may be an underlying cause, though the exact mechanism is unclear. This process is known as lipotoxicity, and it has been linked with the pathophysiology of insulin resistance, type 2 diabetes, liver disease, atherosclerosis, and cardiovascular disease [6]–[9]. Interestingly, one of the proposed anti-lipotoxic strategies is the promotion of fat oxidation through exercise [5].

Food intake and energy expenditure are precisely modulated by specific sets of neurons placed in an area of the brain called the hypothalamus, which comprises the major portion of the ventral part of the diencephalon. The hypothalamus is organized in anatomically-defined neuronal clusters, called nuclei, forming interconnected neuronal circuits via axonal projections. The hypothalamus receives multiple inputs of information as diverse as the sensory experience of eating, the process of ingestion, absorption, metabolism and levels of energy storage. Thus, hypothalamic neurons respond to peripheral nutrients, such as glucose and fatty acids, and hormones, such as leptin, ghrelin and insulin, by modifying the synthesis of orexigenic (feeding-promoters) or anorexigenic (feeding-inhibitors) neuropeptides and then adjusting feeding to the body's nutritional energy demands. When energy intake surpasses expenditure, the expression of orexigenic neuropeptides (such as agouti-related protein (AgRP), and neuropeptide Y (NPY)) diminishes and the expression of anorexigenic neuropeptides (such as cocaine and amphetamine-regulated transcript (CART) and proopiomelanocortin (POMC)) increases. Reverse changes occur when energy expenditure exceeds intake. The impairment of this precise homeostatic system elicits hyperphagia, obesity, and type 2 diabetes [10],[11].

A typical problem associated with failure of the central mechanism governing energy balance is the development of resistance to peripheral signals, such as insulin and leptin [12]–[15]. Although it is well known that some of the molecular mechanisms leading to insulin and leptin resistance involve alterations in key molecules of their intracellular signaling pathways, such as signal transducer and activator of transcription 3 (STAT3), suppressor of cytokine signaling 3 (SOCS3) and protein tyrosine phosphatase 1B (PTP1B) [12],[14],[15], why and how central insulin and leptin resistance are induced by overnutrition is not completely understood. Current evidence has demonstrated that one of the pathological mechanisms of leptin resistance is derived from impairment of endoplasmic reticulum (ER) function, a process known as ER stress and the associate unfolded protein response (UPR) (Figure 2).

**Figure 2:**
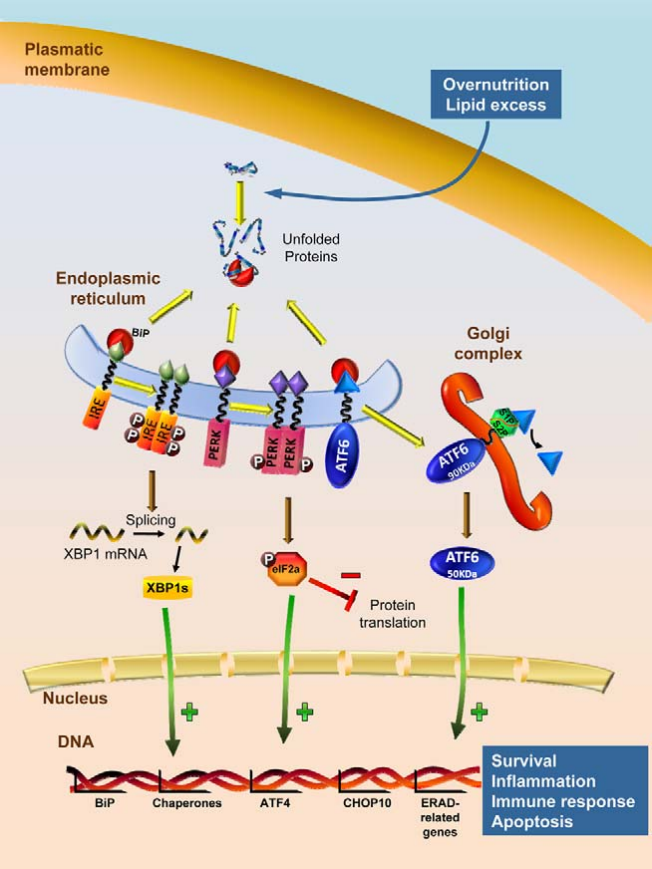
ER stress and UPR signaling pathway. Under optimal nutrient surplus of nutrients, the chaperone BiP remains associated to the luminal surface of ER with three UPR transducer proteins (ER stress sensors: IRE1, PERK, and ATF6), maintaining them inactive. Overnutrition and lipid excess (as well as other insults, such as hypoxia, radiation, oxidative stress, viral infections, etc.) lead to impairment of normal protein folding in the ER, resulting in accumulation of unfolded proteins. In this situation, BiP preferentially binds the misfolded proteins, liberating the ER stress sensors. Dimerization and auto-phosphorylation of IRE1 triggers its endoribonuclease activity to induce cleavage of XBP1 mRNA to its spliced form XBP1s, which upregulates genes encoding chaperones and genes encoding for proteosome machinery, controlling ER-associated degradation (ERAD). PERK is activated by homodimerization and auto-phosphorylation leading to phosphorylation eIF2α, which results in attenuation of general protein translation and increased transcription of ATF4, which will induce transcription of pro-apoptotic genes such as CHOP10. ATF6 released from BiP is translocated to Golgi complex where it is cleaved by S1P/S2P proteases, releasing the 50 KDa domain, that acts as a transcription factor of gene-encoding chaperones including BiP. The overall effect of these events is an adaptive program comprising four different sequential responses, depending on the grade and persistence of the stimulated ER stress cell: (1) a transcriptional and translational attenuation, which reduces synthesis of new proteins preventing further accumulation of misfolded proteins; (2) upregulation of genes encoding ER chaperones to increase protein folding in the ER and prevent aggregation of unfolded proteins; (3) if stimuli persists, proteosome machinery is increased by transcription induction of its genes improving ERAD; and (4) if all of its processes do not rescue the cell from the ER stress and if ER stress inducers persist, genes encoding cell death and apoptosis of the cell will be induced. ATF4 and 6: activating transcription factor 4 and 6; BiP: binding immunoglobulin protein, also known as GRP78, glucose regulated protein 78 KDa; CHOP10: C/EBP homologous protein, also known as DDIT3, DNA-damage inducible transcript 3; eIF2α: eukaryotic initiation factor 2α subunit; IRE1: inositol requiring enzyme 1; PERK: PKR-like ER kinase, also known as dsRNA-dependent protein kinase like ER kinase; S1P/S2P: site-1, site-2 proteases; XBP1: X-box binding protein 1; XBP1s: X-box binding protein 1, spliced.

The ER is a sophisticated luminal network in which protein synthesis, maturation, folding, and transport take place [16]–[18]. The term ER stress refers to the alterations of the protein folding functionality of the ER, which leads to activation of a complex signaling network termed the UPR that results in a coordinated transcriptional response associated to attenuation of protein synthesis, upregulation of ER folding machinery (a type of proteins called chaperones), and degradation of irreversibly misfolded proteins(Figure 2). If this UPR adaptive response is not sufficient to resolve the protein-folding defect, ER impairment can lead to cell dysfunction and, ultimately, to apoptotic cell death [18]. Previous studies have demonstrated that ER stress and activation of UPR pathways play a major role promoting obesity-induced insulin resistance and type 2 diabetes in peripheral tissues. For example, inflammation, free fatty acids (FFAs), and hyperglycemia in pancreatic β cells elicit activation of the UPR, leading to decreased insulin mRNA expression and inhibition in the insulin signaling [19]–[22]. Data produced in the last two years have demonstrated that obesity and overnutrition also induce hypothalamic ER stress [23]–[26]. Specifically, overnutrition activates hypothalamic IKKβ/NF-κB, a well-known mediator of metabolic inflammation, which elicits ER stress (which also promotes IKKβ/NF-κB) and initiates UPR signaling pathways in the hypothalamus, which in turn directs to inhibition of leptin receptor signaling pathway (STAT3, SOCS3, and PTP1B) and insulin and leptin resistance [23]–[25]. Remarkably, the same studies demonstrate that genetic inactivation of IKKβ/NF-κB signaling, or pharmacological interventions that improve protein folding (chemical chaperones), recover leptin and insulin signaling and subsequently normalize food intake and decrease body weight [23]–[26].

Exercise is believed to be a keystone of the treatment for obesity. In fact, physical activity has long been reported to reduce body weight and adiposity, increasing energy expenditure and improving the overall metabolic status of obese patients [5],[27],[28]. In keeping with this evidence, current data have highlighted that exercise improves hypothalamic insulin and leptin sensitivity in rats [29],[30], although the molecular underpinnings of this effect remain unclear. In this issue of *PLoS Biology*, José B.C. Carvalheira and colleagues move our neurobiological understanding significantly forward by suggesting a novel mechanism by which exercise exerts its beneficial effects on energy balance through a hormonal effect at the level of the hypothalamus [31]. They used two different obese models, diet-induced obesity (DIO) rats and leptin-deficient *ob/ob* mice, to show that exercise restores food intake, insulin, and leptin sensitivity to the levels of lean (control) animals. These effects are linked to: (1) normalized expression of hypothalamic neuropeptides modulating feeding (such as NPY and POMC) and (2) improvement of hypothalamic leptin and insulin signaling pathways, all of them severely impaired in obese animals. Of note, authors demonstrate that the molecular mechanism under this action is the reduction of hypothalamic IKKβ/NF-κB activation and ER stress (both of them severely increased in obese animals) through a novel hypothalamic mechanism involving increased serum and hypothalamic expression of the pro-inflammatory cytokine interleukin-6 (IL-6), which induces the expression of the anti-inflammatory cytokine interleukin-10 (IL-10), also in the hypothalamus (Figure 3) [31].

**Figure 3:**
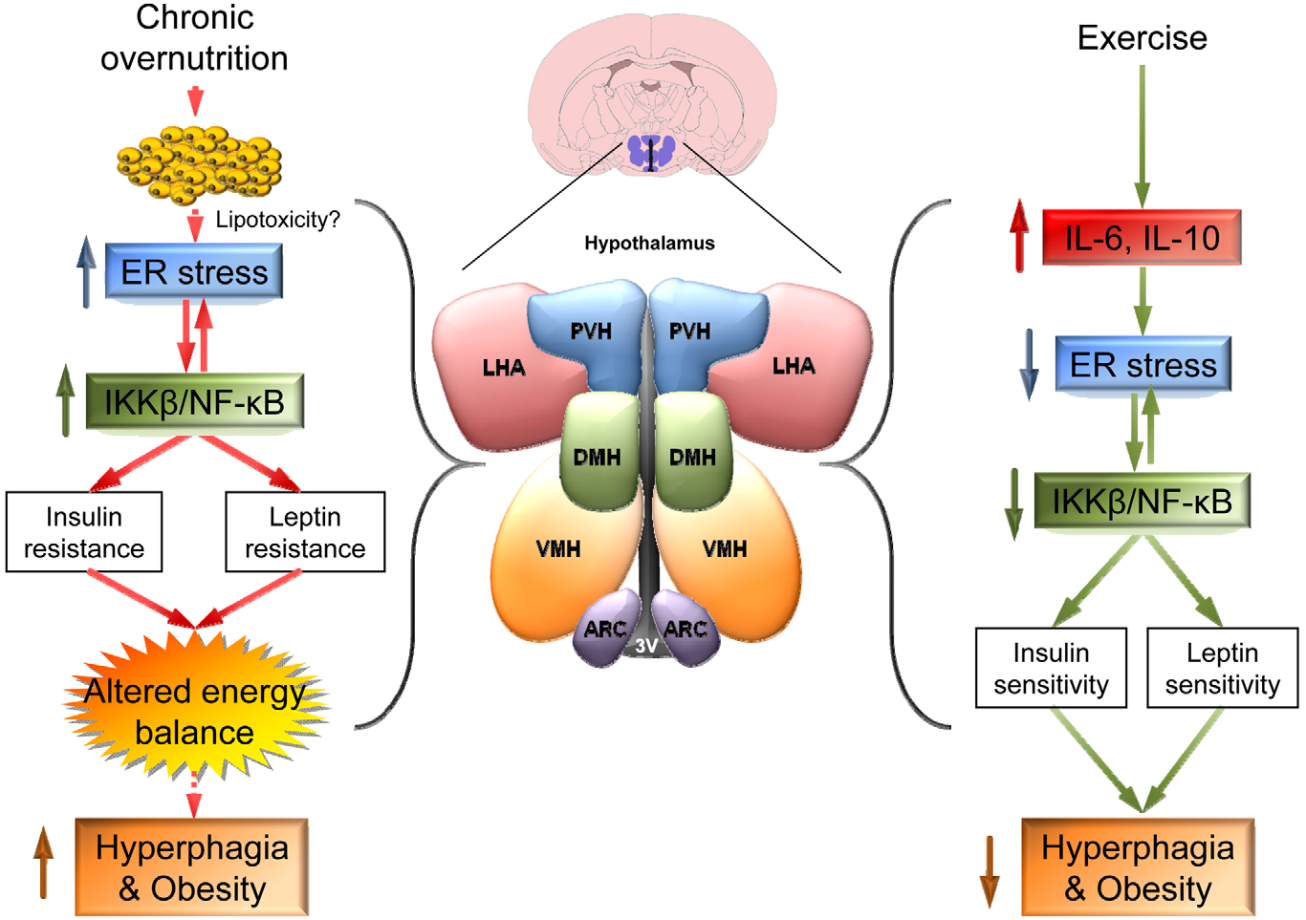
Exercise and ER stress in hypothalamus. Overnutrition increases hypothalamic activation of IKKβ/NF-κB and ER stress (they enhance each other), which leads to insulin and leptin resistance in the hypothalamus, hyperphagia, and obesity. Exercise reduces hypothalamic IKKβ/NF-κB activation and ER stress through a mechanism involving IL-6 and IL-10. As a result of this effect, insulin and leptin sensitivity, and consequently food intake, are restored. Whether hypothalamic lipotoxicity contributes to increased activation of IKKβ/NF-κB and ER stress will require further investigation. 3V: third ventricle; ARC: arcuate nucleus of the hypothalamus; DMH: dorsomedial nucleus of the hypothalamus; IKKβ/NF-κB: inhibitor of nuclear factor kappa-B kinase subunit beta/nuclear transcription factor kappa-B; LHA: lateral hypothalamic area; PVH: paraventricular nucleus of the hypothalamus; VMH: ventromedial nucleus of the hypothalamus.

The significance of these data is given by several novel findings. First of all, although physical exercise was already a key strategy for the prevention of obesity and related diseases [27],[28], this study provides new evidence showing how exercise exerts a direct impact by improving the metabolic and cellular status of the hypothalamus in the brain. Exercise reduces hypothalamic IKKβ/NF-κB and ER stress and improves insulin and leptin action in an IL-6–dependent manner, which consequently decreases hyperphagia and body weight. Secondly, the effect of exercise seems to be restricted to obese animals since, in absence of obesity, it does not induce significant changes either in feeding or body weight or biochemical and cellular parameters. Finally, in resting conditions the treatments with either IL-6 or IL-10 are able to blunt the hyperphagic response, as well as the leptin and insulin resistance, promoted by pharmacological administration of ER stress inducers (such as the drug thapsigargin, which interferes with Ca^+2^ balance), in both normal and genetic models of cytokine deficiency (such as toll-like receptor 4, Tlr4, deficient mice). These data suggest that the IL-6-IL-10-IKKβ/NF-κB axis is a potential therapeutic target for the treatment of obesity [31]. In this sense, recent evidence has shown that IL-6 treatment improves insulin sensitivity [32] and that IL-6 is also released from the brain during long-term exercise in humans [33]. Overall, these data reveal new pathways and provide novel challenges for the understanding of energy balance and the treatment of obesity., Bearing in mind that lipotoxicity is one of the key mechanisms leading to ER stress in peripheral tissues [6]–[9] and the main role of hypothalamic lipids in the regulation of energy balance [9],[34], it would be crucial to assess whether alterations (genetic or diet-induced) of hypothalamic lipid metabolism could lead to lipotoxicity and subsequently to ER stress-induced obesity (Figure 3). Additionally, in order to establish possible therapeutic targets, the effect of exercise on other ER stress models should be examined. In their study, Carvalheira and colleagues used thapsigargin as ER stress inducer [26],[31]; alternative drugs, such as tunicamycin (which interferes with protein glycosylation) and brefeldin A (which impairs ER-Golgi vesicular transport) [23]–[25], or ideally genetic models will provide new cues about the effect of exercise on hypothalamic ER stress.

In summary, the new study of Carvalheira and colleagues adds further confirmation that hypothalamic ER stress is an important pathogenic mechanism leading to obesity. It provides a novel integrative framework to understand the deleterious metabolic effects of overnutrition and, more importantly, provides a good rationale for the indication of exercise to obese patients. Moreover, it presents a “scientific evidence” to support the famous Latin citation of the Roman poet Juvenal, “*Mens sana in corpore sano*,” and that physical exercise is a key factor to maintain us and our brains in a healthy state.

## Abbreviations

ER: endoplasmic reticulum
ERAD: endoplasmic reticulum-associated degradation
IKKβ/NF-ΚB, inhibitor of nuclear factor kappa-B kinase subunit beta/ nuclear transcription factor kappa-B; IL-6: interleukin-6: IL-10, interleukin-10
NPY: neuropeptide Y
POMC: proopiomelanocortin
PTP1B: protein tyrosine phosphatase 1B
SOCS3: suppressor of cytokine signaling 3
STAT3: signal transducer and activator of transcription 3
UPR: unfolded protein response
WAT: white adipose tissue
